# Electrospray deposition of polymer thin films for organic light-emitting diodes

**DOI:** 10.1186/1556-276X-7-52

**Published:** 2012-01-05

**Authors:** Wontae Hwang, Guoqing Xin, Minjun Cho, Sung Min Cho, Heeyeop Chae

**Affiliations:** 1 School of Chemical Engineering, Sungkyunkwan University (SKKU), Suwon, 440-746, South Korea; 2 SKKU Advanced Institute of Nanotechnology (SAINT), Sungkyunkwan University (SKKU), Suwon, 440-746, South Korea

**Keywords:** organic light-emitting diodes, electrospray, polymer, conformation.

## Abstract

Electrospray process was developed for organic layer deposition onto polymer organic light-emitting diode [PLED] devices in this work. An electrospray can be used to produce nanometer-scale thin films by electric repulsion of microscale fine droplets. PLED devices made by an electrospray process were compared with spin-coated ones. The PLED device fabricated by the electrospray process showed maximum current efficiency of 24 cd/A, which was comparable with that of the spin-coating process. The electrospray process required a higher concentration of hole and electron transport materials in the inks than spin-coating processes to achieve PLED maximum performance. Photoluminescence [PL] at 407 nm was observed using electrosprayed poly(*N*-vinyl carbazole) films, whereas a peak at 410 nm was observed with the spin-coated ones. Similar difference in peak position was observed between aromatic and nonaromatic solvents in the spin-coating process. PLED devices made by the electrospray process showed lower current density than that of spin-coated ones. The PL peak shift and reduced current of electrosprayed films can therefore be attributed to the conformation of the polymer.

## Introduction

During the last two decades, intense academic and industrial research has been devoted on organic light-emitting devices [OLEDs] due to their potential applicability to flat panel displays and solid-state lighting [1-3]. Small areas of less than 5-in. devices have been commercialized recently. Currently, all commercial processes adopt vacuum evaporation processes for both organic and metal layer formations. However, vacuum evaporation processes are significantly limited in large-area processing as well as hardware cost and require a considerable material. These limitations and drawbacks prevent OLEDs from being applicable to large-area device fabrication. Much effort has been made for solution process development that can form nanoscale-thin organic films with a large area while minimizing material wastes. A laboratory-scale spin-coating process is one of the most well known and widely used solution processes. Various solution printing techniques have been developed such as ink-jet printing, nozzle printing, screen printing, gravure printing, and so on. Thin-film transfer process, organic vapor deposition, and blade coating are also being developed as large-area, cost-effective alternatives [4-10]. However, challenges remain relating to good uniformity over a large-area and multilayer formation without buffer or cross-linking materials for commercial development of organic displays and lighting devices.

Recently, electrospray process has gained much attention as a solution process for organic and inorganic thin films, and a few research groups have reported applications to organic device fabrications [11-13]. In the electrospray process, a liquid flow is injected into the nozzle with an electric field applied between the nozzle tip and ground plate, and microscale monodisperse fine droplets are generated due to repulsion forces between like charges in the drops. The size of droplets can be controlled by adjusting the flow rate and electric field applied to the injection nozzles and substrates, and the diameter of the droplets can be as small as several hundred nanometers in scale [14-16]. The electrospray process with vapor treatment has been applied to organic photovoltaic fabrication, while comparable power conversion efficiency has been reported with the spin-coating process [13]. Additionally, applicability of the electrospray process to organic thin films in OLEDs was demonstrated in small-scale devices [12].

In this work, we demonstrated nanoscale-thick organic thin films using the electrospray process as a solution process alternative. Polymer LEDs [PLEDs] were fabricated and compared with those made by the spin-coating process. The electrospray process can be considered as an effective process for patterning, multilayer stacking, and continuous processing of organic thin films.

## Experimental details

For PLED fabrication, we used a blended solution of poly(*N*-vinyl carbazole) [PVK], 2-(4-biphenylyl)-5-(4-tert-butylphenyl)-1, 3, 4-oxadiazole [PBD], *N, N*'diphenyl-*N, N*'-Bis(3-methylphenyl)-[1, 1-biphenyl]-4, 4'-diamine [TPD], and tris(2-(4-tolyl) phenylpyridine) iridium [Ir(mppy)_3_] dissolved in chlorobenzene [CB], dichlorobenzene [DCB], or a mixture of CB and 1, 2-DCB. Two different types of inks were formulated as shown in Table 1. PLED devices were fabricated with a ratio of PVK/PBD/TPD/Ir(mppy)_3 _= 61:24:9:6 (ink 1) for both the spin-coating and electrospray processes. The PBD/TPD ratio was increased for the electrospray process. CB is best for the spin-coating process among the solvents mentioned earlier, and DCB addition is required for the electrospray process.

**Table 1:** Ratio of the PLED ink

	**Ink 1**^**a**^	**Ink 2**^**a**^
PVK	61	41.5
PBD	24	41.5
TPD	9	14.8
Ir(mppy)_**3**_	6	4.2

^a^Each figure means weight percent of materials in the solution, and the main difference between Ink 1 and Ink 2 is the ratio of hole and electron transport materials. PVK, poly(*N*-vinyl carbazole); PBD, 2-(4-biphenylyl)-5-(4-tert-butylphenyl)-1, 3, 4-oxadiazole; TPD, *N, N*'diphenyl-*N, N*'-Bis(3-methylphenyl)-[1, 1-biphenyl]-4, 4'-diamine; Ir(mppy)_3_, tris(2-(4-tolyl) phenylpyridine) iridium.

**Figure 1:**
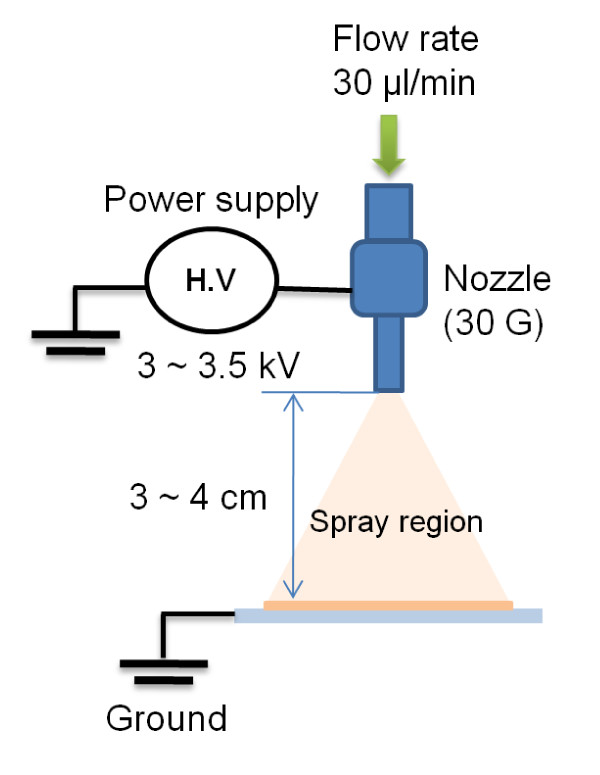
**Schematic description of the electrospray process used for PLED fabrication**.

The layer structure of the devices was as follows: indium tin oxide [ITO]/poly(3, 4-ethylenedioxythiophene) poly(styrenesulfonate) [PEDOT:PSS]/active layer/metal cathode. A hole-injection PEDOT:PSS layer of 30-nm thick was first spin-coated on pre-cleaned ITO substrates and then baked at 120°C for 20 min. About 80-nm-thick emissive layer [EML] was formed by electrospray or spin-coating process and then annealed at 80°C for 30 min. A schematic diagram of the experimental setup for the electrospray process is shown in Figure 1. For the electrospray process, the blend ink was injected through the nozzle at a rate of 30 μl/min, and about 3 kV was applied to break the meniscus formed at the tip of the nozzle. The distance between the tip of the nozzle (150 μm in diameter) and the substrate was maintained at 3 to 4 cm. During spraying, the nozzle and substrate were fixed, and the thickness of EML was controlled by varying the deposition time. For the comparative spin-coating process, the solution was spin-coated at 2, 000 rpm for 20 s. All the experiments were carried out at 20°C to 25°C and a humidity of 30% to 35%. The interlayer and cathode were thermally evaporated on the top of the EML at a pressure of 2 × 10^-5 ^Torr.

All electrical measurements were performed under ambient conditions. Device performance was measured using a source measure unit (2400, Keithley Instruments, Inc., Cleveland, OH, USA) and a luminance meter (CS100, Konica Minolta Sensing, Inc., Sakai, Osaka, Japan). Photoluminescence [PL] spectra were collected with a monochromatized 150-W Xe light source (FP-6200, Jasco International Co. Ltd., Hachioji, Tokyo, Japan), and a wavelength of 335 nm was used for analysis of PL excitation. The thickness and roughness of PVK films were determined by a surface profiler (Alpha-Step, KLA-Tencor Corporation, Milpitas, CA, USA) and by atomic force microscopy (Digital Instruments, Santa Barbara, CA, USA), respectively.

## Results and discussion

**Figure 2:**
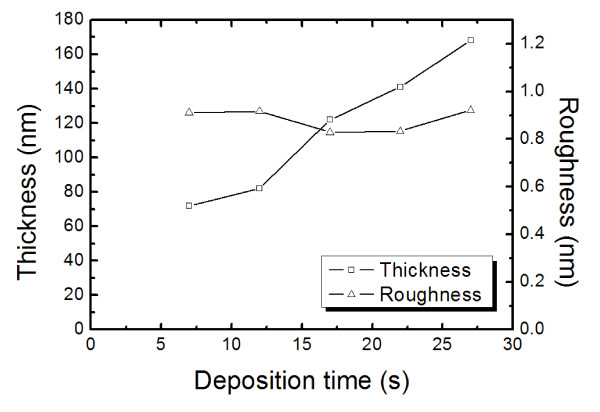
**PVK film thickness and roughness as a function of deposition time**.

In electrospray deposition, it is critical to choose proper solvents to make fine droplets and uniform thin films. Evaporation rate of the solvent, which is determined by its boiling point, is an important factor to consider. Dielectric constant is another crucial physical property of solvents in determining the size of droplets and droplet dispersion. Further, high dielectric constant and high boiling point are preferred in electrospray processing. Specifically, 1, 2-DCB (dielectric constant [*ε*] = 10, boiling point = 180°C) is added to CB (*ε *= 5, boiling point = 130°C) [17] to balance solubility and droplet control. The concentration of droplets at the time of deposition is then controlled by managing the droplet size. Droplets containing little solvent or almost dry particles can be deposited when a low-boiling point solvent is used at a slow flow rate. This mode of operation is called the dry mode, and a few groups have reported a dry-mode operation [18]. In this work, droplets containing a rather large fraction of solvents were transferred to substrates, and therefore, the films required time for solvent evaporation after the deposition. As shown in Figure 2, 70- to 150-nm-thick light-emitting organic layers with about 1-nm surface roughness were formed by the electrospray process in this work.

**Figure 3:**
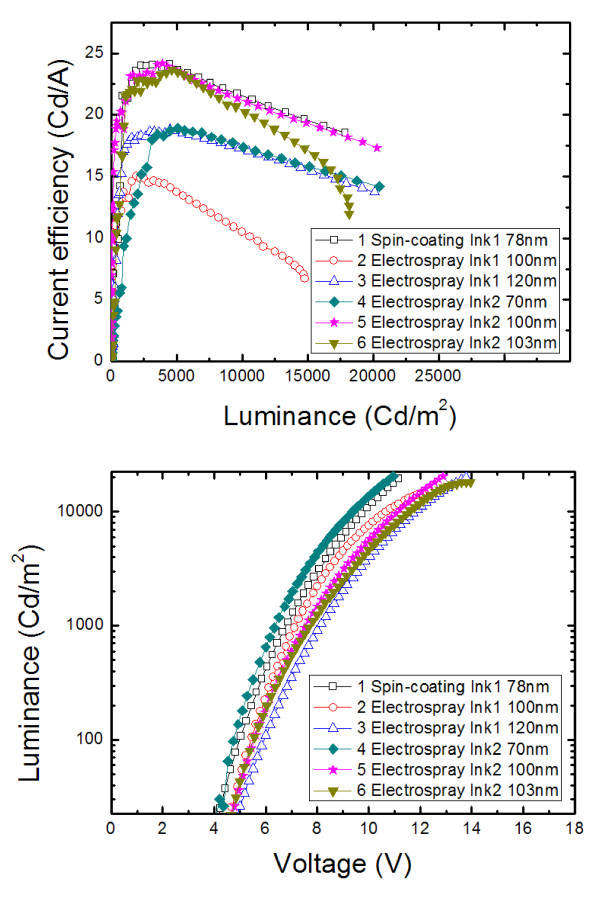
**Device performance of the devices made by spin-coating or electrospraying**. The thicknesses of EML were as follows: (1) 78, (2) 100, (3) 120, (4) 70, (5) 100, and (6) 103 nm.

To investigate the characteristics of organic light-emitting layers made by the electrospray deposition process, we adopted organic light-emitting materials of PVK as a host polymer with Ir(mppy)_3 _added as a dopant. Further, TPD and PBD were added to facilitate a hole and electron transport. We performed experiments with our standard composition, ink (1) in Table 1, and the device made with the spin-coating process with ink (1) is shown with sample 1 in Figure 3. When we used the same ink (1) in the electrospray process, the current efficiency significantly decreased by up to 38% (sample 2). We believe from the cause was an imbalance in charge transport in the device, and we were able to achieve similar performance by formulating ink (2) with increased hole and electron transport materials as shown with sample 5 in Figure 3. Samples 5 and 6 show the effects of the solvent in the electrospray process. Better device performance was achieved with a solvent mixture of CB/DCB = 5:3. DCB helps maintain a stable and uniform spray, but too much DCB lowers device performance since it cannot be removed sufficiently during the annealing process due to its high boiling point. This result indicates that solvent optimization can regulate film and device performance made by the electrospray process. The summary of device performances is in Table 2.

**Table 2:** Summary of device performances

Device	Process	Ink	Solvent	EML (nm)	Cd/A (max)	Lm/W (max)
1	Spin-coating	Ink 1	CB	78	24	10
2	Electrospray	Ink 1	CB/DCB (5:3)	100	15	6
3	Electrospray	Ink 1	CB/DCB (5:3)	120	18	6.6
4	Electrospray	Ink 2	CB/DCB (5:3)	70	18	7.5
5	Electrospray	Ink 2	CB/DCB (5:3)	100	24	9.2
6	Electrospray	Ink 2	CB/DCB (1:1)	103	23	8.6

EML, emissive layer; CB, chlorobenzene; DCB, dichlorobenzene.

**Figure 4:**
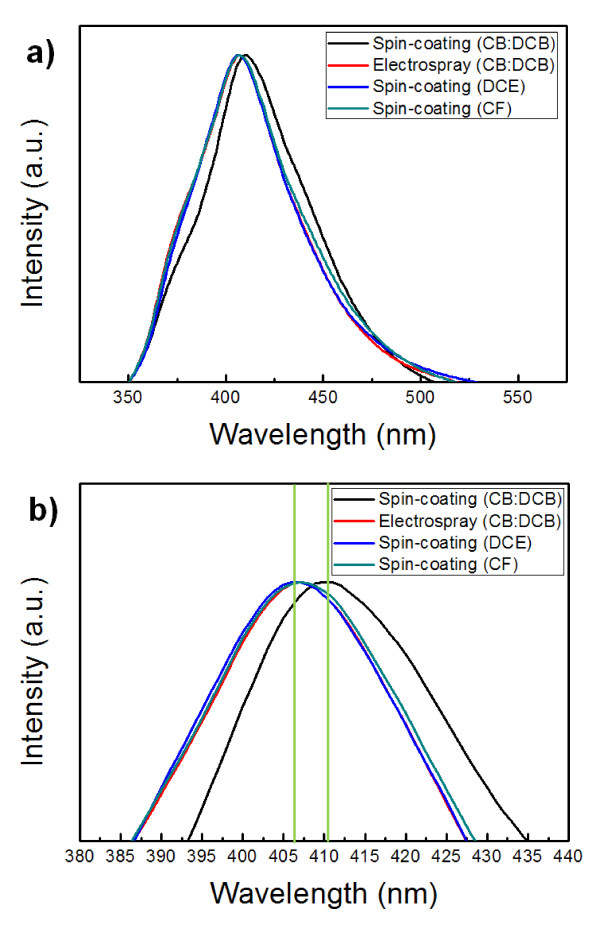
**PL spectra of PVK films spin-coated or electrosprayed from the solvents (CB/DCB, DCE, or CF)**. (**a**) Normal view. (**b**) Enlarged view of (a). All spectra are normalized to their maximum value, and the excitation wavelength was fixed at 335 nm.

**Figure 5:**
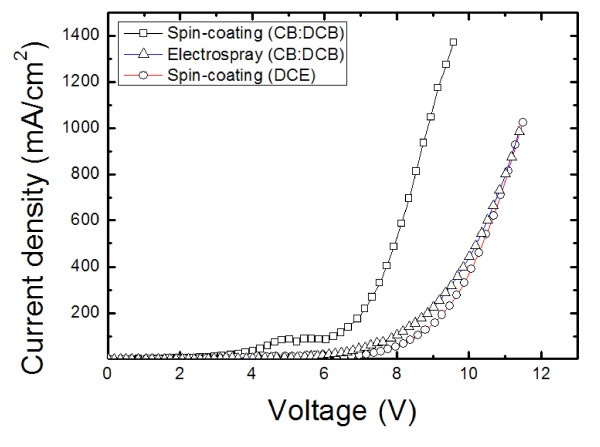
**Current density-voltage curve of PVK devices using spin-coating and electrospray deposition (ITO/PEDOT:PSS/PVK/CsF/Al)**. The thin films using CB/DCB showed a better current density under the same conditions.

To investigate the difference between electrospray and spin-coating processes, PVK thin films were formed by two different processes, and their electric and PL characteristics were investigated. As mentioned earlier, development of a proper solvent is an important step in the electrospray process. Accordingly, we also investigated the effects of solvents on electric and PL film characteristics. Figure 4 shows the effects of solvents and process method on PL characteristics. We chose two different types of solvents: an aromatic solvent which comprised a mixture of CB and DCB and non-aromatic solvents, chloroform [CF] and 1, 2-dichloroethane [DCE]. The mixture of CB and DCB was selected as it was optimum for our device structure and process. The PL peaks of the thin films were observed at 407 nm with CF and DCE and at 410 nm with the aromatic solvent mixture. Using the same aromatic solvent, PVK films processed by the electrospray process showed PL peaks at 407 nm, while those made by spin-coating showed PL peaks at 410 nm. Qian et al. [19] reported a peak shift of PVK due to polymer conformation caused by the solvent effect. We believe that a similar polymer conformation effect results not only from the solvent, but also from the processing method as shown in this work. Figure 5 shows the current-voltage characteristics of the PVK film using different solvents and processing methods. At the same thickness, the thin films using the aromatic solvent (mixture of CB and DCB) showed a higher current density than those processed with the DCE nonaromatic solvent. In terms of processing methods, the PVK film processed with the electrospray method showed a lower current density than that of spin-coated ones. These results suggest that polymer conformation of PVK thin films was affected by the processing method as well as by the solvent's molecular structure as also indicated in PL results in Figure 4. Based on these results, we speculate that the properties of thin films made by the electrospray process are similar to those of spin-coated films made with DCE.

## Conclusion

We have demonstrated polymer organic LEDs using an electrospray process as a solution process alternative. Device performance comparable to spin-coating was achieved with the electrospray process, which can be considered as a scalable large-area process alternative. However, the electrospray process requires elaborate choices involving solvents and higher concentrations of hole and electron transport materials in active materials. A high dielectric constant and a high-boiling point solvent like DCB are preferred in electrospray processing. Accordingly, the device based on the electrospray process had better performance when we used the ink having higher concentrations of hole and electron transport materials. The electrospray process can be considered as a viable solution for large-area organic thin-film formation technology in the future.

## Competing interests

The authors declare that they have no competing interests.

## Authors' contributions

WH carried out the overall experiment. GX helped in the PL measurement. MC participated in the device fabrication. SMC and HC gave advice for this work. All authors read and approved the final manuscript.
